# Development and early validation of questionnaires to assess system level factors affecting male partners’ attendance at childbirth in LMICs

**DOI:** 10.1186/s12884-023-05580-y

**Published:** 2023-04-17

**Authors:** Thierry Claudien Uhawenimana, Nicola M. Gray, Heather Whitford, Alison McFadden

**Affiliations:** 1 College of Medicine and Health Sciences, School of Nursing and Midwifery, Department of Midwifery, University of Rwanda, Po. Box: 3286, Kigali, Rwanda; 2 School of Health Sciences, University of Dundee, Dundee, DD1 4HJ Scotland, UK

**Keywords:** Male partners’ attendance at childbirth, Questionnaire design, Delphi study, Birth companions

## Abstract

**Background:**

There is evidence that a woman who receives continuous labour support from a chosen companion can have shorter labour duration, is more likely to give birth without medical interventions, and report a satisfying childbirth experience. These outcomes result from the beneficial effects of emotional and practical support from the woman’s chosen companion, and care provided by health providers. When a woman’s chosen companion is her male partner, in addition to the above benefits, his presence can promote his bonding with the baby, and shared parenthood. However, there may be healthcare system barriers, including organisational, management and individual (staff) factors, that inhibit or restrict women’s choice of companion. There are currently no suitable survey tools that can be used to assess the system level factors affecting the implementation of male partners’ attendance at childbirth in low- and middle- income countries (LMICs).

**Methods:**

We designed two questionnaires to help to address that gap: the Male Partners’ Attendance at Childbirth-Questionnaire for Heads of Maternity Units (MPAC-QHMUs); and the Male Partners’ Attendance at Childbirth-Questionnaire for Maternity Staff (MPAC-QMS). We carried out an extensive review to generate initial items of the two questionnaires. We assessed the content and face validity of the two questionnaires in a three-round modified Delphi study.

**Results:**

The Male Partners’ Attendance at Childbirth-Questionnaire for Heads of Maternity Units (MPAC-QHMUs) focused on organisational and management factors. The Male Partners’ Attendance at Childbirth-Questionnaire of Maternity Staff (MPAC-QMS) focused on individual staff factors. The final MPAC-QHMUs and MPAC-QMS included items which garnered over 80% content relevance according to the experts’ rating. After all three consensus rounds of the Delphi study, 43 items were retained for the MPAC-QHMUs and 61 items were retained for the MPAC-QMS.

**Conclusions:**

The MPAC-QHMUs and the MPAC-QMS may help understanding of barriers affecting male partners’ attendance at childbirth in LMICs in order to devise implementation strategies to enable wider availability and to maximize women’s choices during labour and childbirth. The MPAC-QHMUs and the MPAC-QMS as newly-developed questionnaires require further validation of their acceptability and feasibility in different cultural contexts, and languages.

**Supplementary Information:**

The online version contains supplementary material available at 10.1186/s12884-023-05580-y.

## Introduction

In a bid to enhance overall childbirth experience, the World Health Organization (WHO) recommends that health facilities enable women to have a companion of choice throughout labour and childbirth [1]. In addition, labor companionship is a key WHO recommendation for a safe and dignified birth experience [1]. Birth companions may include doulas; female birth companions, including mothers, sisters, mothers-in-law, other female relatives, female friends; traditional birth attendants [1]; and/or a male partner [2–4]. Unfortunately, research shows that most health facilities, particularly in LMICs, deny women this right [5], which hinders the goal of improving the quality of care given to women and their families during childbirth.

In some western societies such as New Zealand, UK, Sweden, and USA, it has become commonplace for women to be accompanied by their male partners during labour and/or birth [4, 6, 7]. In LMICs, a multi-country community based survey that included 2672 women from Ghana, Guinea, Nigeria and Myanmar found that half of women had companions at any point during childbirth [8]. This survey reported that only in Nigeria, the number of women whose partners had attended childbirth was higher than other countries because out of 240 surveyed women, 47.9% of them were with their male partners during labour. Evidence from individual studies from Ethiopia [9], Myanmar[10, 11],Guatemala [12, 13], China [14], India [15], Nigeria [16], and Brazil [17] reported that male partners’ attendance ranged between 16 and 87%. However, sometimes, the reported percentages only reflected the proportion of men who accompanied their female partners to the place of birth but did not stay to support them. It can be concluded from these studies that male partners’ attendance at childbirth in LMICs is a recent development. However, there is a dearth of current research evidence from LMICS about health facilities’ willingness to enable women’s choice of a male birth companion during labour and/or birth and the feasibility of implementing this practice.

The success of evidence-based practice is driven by the organisational receptive capacity to embrace change [18, 19]. It is also driven by health providers’ attitudes to the proposed innovation and their skills to implement it [18, 19]. Much as women may wish for their male partners’ presence at labour and/or birth, research shows that many public health facilities in LMICs restrict male partners’ attendance at childbirth regardless of the views of the woman [20, 21, 30–34, 22–29]. However, the evidence on the reasons leading to these restrictions remains incomplete. Hence, there is a need to explore factors affecting male partners’ attendance at childbirth from organisational and staff perspectives to understand health facilities’ implementation of this practice.

Whilst there is some literature about fathers’ needs and experiences when they attend childbirth [35–39], the search for suitable survey tools found a dearth of evidence focused on assessing current practice, service level factors, and maternity staff attitudes to male partners’ attendance at childbirth in health facilities globally and more particularly in LMICs. In particular, there is a lack of standardised methods to assess system level factors. Only one tool from high income settings was identified that attempted to address health care providers’ perceptions of male partners’ attendance at childbirth, but, components that may explain providers’ perceptions such as culture, health system, and perceived impact of this practice were missing [40].

The objectives of this study were to construct questionnaires that could be used to collect data about current practice, service level factors (culture, education, training, and structure), and health providers’ views and attitudes regarding the acceptability and feasibility of male partners’ attendance at childbirth, and facilities’ readiness to implement that practice. These questionnaires were designed to help inform implementation strategies to maximize women’s choice of birth companion in LMICs.

## Methods

### Generating items for the initial draft questionnaires

Items for the initial drafts of the questionnaires were developed following an extensive review of literature undertaken during the first author’s PhD project [41]. The review work included an overview of systematic reviews about male partners’ attendance at childbirth and two systematic reviews that focused on factors influencing male partners’ attendance at childbirth in LMICs [41]. Additional items were sourced from relevant items identified during a review of existing tools on fathers’ attendance at childbirth [41].

Two questionnaires were constructed, one targeted at an organisational level and the other at the individual staff members. The *Male partners’ attendance at childbirth: Questionnaire for Heads of Maternity Units (MPAC-QHMUs*) was designed to be administered to the heads of maternity units to collect organisational level information, mainly current practice, facilitators of, and barriers to, male partners’ attendance at childbirth, and the facilities’ readiness to initiate the practice, or sustain it where it was already operational. The *Male partners’ attendance at childbirth: Questionnaire for Maternity Staff (MPAC-QMS)*’ was designed to be administered to maternity staff (nurses, midwives, obstetricians, and other ancillary staff working in maternity units) to collect information about maternity staff practices, perceptions, and attitudes regarding the acceptability and feasibility of male partners’ attendance at childbirth. It was important to collect data at an organisational level to assess the extent to which organisations develop policy to encourage the use of evidence-based practice in providing care, and at an individual staff level to assess whether individual staff were ready to embrace the change in practice; any disconnect between the two is likely to result in a failure of implementation [42].

We drafted an initial pool of items in three stages: 1) elicitation of the section headings and assigning items for each; 2) compiling the first draft; and 3) refining and arranging items for each questionnaire.

First, section headings constituting the indicators of each questionnaire were identified from the systematic reviews [41]. After setting up section headings for each questionnaire, the next exercise involved collecting relevant statements for each section heading from primary studies included in the reviews s.

The next stage involved drafting items for each questionnaire’s section headings based on the statements. An appropriate response option for each item was added. Response options included yes/no, Likert scale or frequency response options. A pool of items for each questionnaire to be assessed by the experts was compiled.

The drafting process entailed a team approach and considered best practice in questionnaire development; mainly comprehension, readability, clarity, relevance, length of questionnaire, and topic sensitivity [43–46].

The generation of items for the two questionnaires followed the rational approach to development of scales and questionnaires [47]. The rational method is a non-theoretical approach to questionnaire design guided by face and content validity and is based on the knowledge of experts rather than known theories of scale construction [47–49]. The item review procedure is carried out to assure face and content validity [47, 49]. During this study, the development and early validation of the two questionnaires was achieved through the conduct of a modified Delphi study. Modified Delphi study is a group-based approach that may be used to obtain consensus from a group of experts through administering a series of questionnaires with iterative controlled opinion feedback [50, 51]. The modified Delphi was chosen for this study because it uses fewer than three rounds and can be administered using email [51, 52]. In addition, since items were drafted from the literature review, the modified Delphi was suitable to meet the purposes of early validation of the developed items by the expert panel and generate additional items from the panel’s comments.

### Face and content validity of the MPAC-QHMUs and MPAC-QMS

The initial drafts of the questionnaires, excluding the background information section, had 47 organisational level items (MPAC-QHMUs) and 83 individual staff level items (MPAC-QMS).

**Fig. 1:**
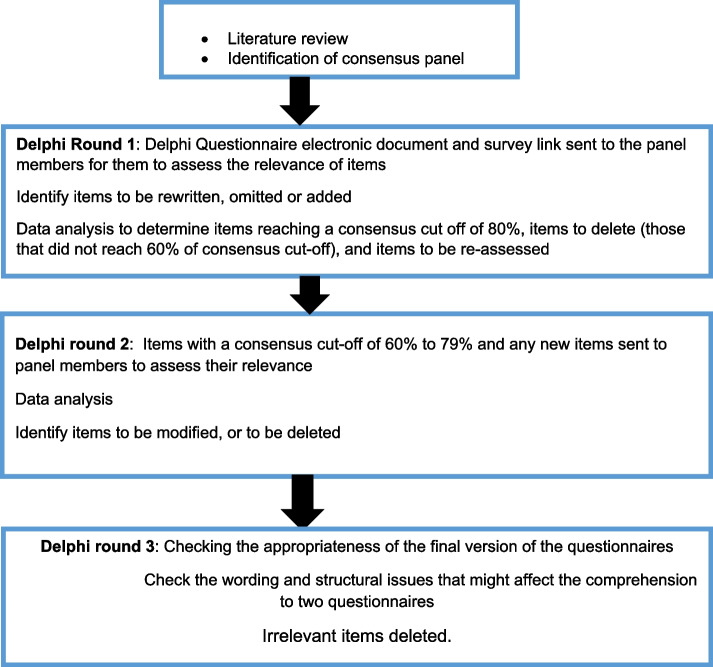
Purpose and activities done in each Delphi round

The face and content validity of the two newly-developed questionnaires were assessed using a Modified Delphi Survey [51, 53]. Face validity involved assessing the extent to which the content of the two questionnaires were sufficiently comprehensive [44, 54]. Content validity involved the assessment of the relevance of items to the contextual factors that may influence men’s presence at labour and/or birth in LMICs [44]. Steps that guided the evaluation of the questionnaires’ relevance are displayed in Fig. 1 below:

### Expert panel sampling and recruitment

An expert panel was convened that included people who were informed advocates of male partners’ attendance at childbirth in LMICs through their working experience, research, and teaching activities. A list of experts was generated by identifying representatives of organisations, institutions, and editorial boards of relevant academic and professional journals. We aimed for a multi-country panel to capture a wide range of experience and expertise relating to male partners’ attendance at childbirth. A multidisciplinary group of participants from nursing and midwifery practice, education, obstetrics, maternal and child health, policy-making, and for some, fathers’ involvement in maternal health in LMICs was purposively recruited.

Assuming a response rate of 35%, inviting 34 people to the panel was needed to account for attrition during the survey rounds to achieve a sample size of 12 [51, 53, 55]. A minimum of 12 respondents would be sufficient to enable consensus to be achieved [56]. Recruitment of participants was completed through email invitation accompanied by a participant information sheet and a copy of the ethics approval letter. A follow-up email was sent to participants who did not respond to the first email within two weeks. It was assumed that participants who did not respond to the second invitation did not wish to participate in the study.

### Data collection

A modified Delphi survey that involved three rounds (earlier illustrated in Fig. 1) was completed through electronic communication using email and Jisc online survey service provided by the University of Dundee.

In December 2018, participants who agreed to take part were invited to complete the first round of the Delphi survey. The email contained the questionnaire as an attachment and a link to a web version of the survey.

Experts were initially presented with the pool of 130 items, and were asked to 1) judge their relevance from a choice of not relevant, somewhat relevant, quite relevant, and very relevant; 2) assess the clarity of the questions being asked to provide suggestions for improvement where needed; and 3) propose additional items if gaps were identified.

Participants were given three weeks to complete and return the questionnaires, with a reminder email sent after two weeks. After the second reminder, those who had not returned the survey were classified as non-respondents and no further follow-up was made.

Returned questionnaires were analysed to compute participants’ consensus agreement. For each questionnaire, items which reached 80% or more agreement (‘quite relevant’ and ‘very relevant’ response options combined) were revised if needed and were not sent out for round two. Items that scored 60% to 79% consensus were revised following the panel’s’ feedback where necessary and were re-assessed in round two. New items were drafted from participants' suggestions and were included in the round two questionnaire [57]. Items that did not reach 60% consensus were deleted.

### Delphi round 2

The purpose of round two was to assess the relevance of items with a consensus cut-off of 60% to 79% and new items that emerged from Delphi round 1. Round two was launched in January 2019. All participants who completed Delphi round 1 were invited to assess the relevance of the items sent to them in Delphi round 2. The same assessment procedure used to garner consensus on the relevance of items in round one also applied to round two of this study. Participants were given three weeks to return the survey with a reminder email after two weeks. Items which did not reach a cut-off of 80% were discarded.

### Delphi round 3

The purpose of round three was to check the clarity of items, and identify any items from the previous rounds that were not relevant to the topic under study.

In March 2019, round three was launched. Participants who completed the previous two rounds were sent an email inviting them to access an online link to the prototype of the MPAC-QHMUs and MPAC-QMS. Participants were asked to read the items proposed for the two questionnaires and identify any wording, clarity, or structural issues that would affect comprehension by the target population. In this round, participants were given two weeks to return the survey with a reminder email after one week. No follow-up emails were sent to participants who did not return the survey after the reminder.

### Data analysis

After each round, participants’ responses were entered into SPSS version 22.0 software. Descriptive statistics were applied to analyse and report demographic information about the expert panel. Frequency distributions were computed to identify patterns of agreement regarding the relevance of items.

Consensus was defined as the percentage of agreement among participants on the item’s relevance. An item was retained if at least 80% of participants rated it as ‘quite relevant’ or ‘very relevant’ [58]. Items that achieved 80% agreement but needed some modifications to aid clarity were amended and retained.

Comments for the improvement of some items submitted by the participants were considered and changes made as needed.

### Ethics, informed consent, and data security

This study was approved by the University of Dundee, School of Health Sciences/ Research Ethics Committee (Application Number: 2018014 Uhawenimana). Information about the study was provided to participants in the Participant Information Sheet. Participants confirmed their consent to voluntary participation in the study by completing the questionnaires. Data was processed in accordance with the University of Dundee data protection policy.

## Results

### Panel characteristics

The invitation to take part in the study was sent to 34 people. Half of them (*n* = 17) responded to the email and 15 agreed to participate resulting in a response rate of 44%. Round one was completed by 12 participants, round two by nine people, and round three by eight people.

The expert panel after round one comprised six people from Rwanda, three from Uganda, one from Canada, one from Belgium, and one expert in fatherhood research from the UK. Nine participants were female and three were male. Nine participants had a Masters’ degree and three held Bachelors’ Degree at the time of the study. Participants had either an educational or professional background in midwifery (*n* = 2), nursing and midwifery education (*n* = 6), obstetrics (*n* = 1), research in maternal and neonatal health (*n* = 2), and fatherhood studies (*n* = 1). Ten participants held clinical, teaching, and research roles. Two occupied decision-making positions about safe motherhood policy and had research experience in maternal health and male involvement.

### Results of delphi survey round 1

After round one, out of 47 items for the MPAC-QHMUs sent for assessment, 25 that achieved 80% cut-off to be retained required some editing and six were deleted (see supplementary information N^o^: 1). After this round, nine items were added (Table 1). For the MPAC-QMS, out of 83 items sent for assessment, 14 items were modified, six items had a consensus cut-off between 60 and 79% and were reassessed in round two, seven were deleted (see supplementary information N^o^: 2), and two new items were added (Table 2).

**Table 1:** MPAC-QHMUs proposed new items after round one

*Proposed question/item*	*Section where proposed question/item may fit*
How many birth companions does your facility allow to be with the woman during childbirth?	Section One: Health facilities' current practice of facilitating fathers' attendance at labour and/or birth
Does your health facility request the woman’s consent before permitting a companion of her choice to attend childbirth?	Section One
Does the policy or guideline describe the father’s role when attending labour and birth?	Section One
If it is the woman’s choice, does your health facility currently allow fathers to attend delivery?	Section One
Under what condition (s) do you permit fathers to attend labour and/or birth? Response options included: 1. If it is the woman’s choice 2. If the father wishes to attend childbirth 3. The father is obliged to be present at labour and delivery once he arrives at the health facility 4. Only when the birth is expected to be normal 5. If there is a likelihood that the woman will develop complications 6. Only when the woman is to be transferred to another facility 7. Caesarean Section	Section One
Our health facility does not encourage fathers’ attendance at childbirth because they can get in the way of maternity staff	Section Two: Factors determining whether or not health facilities encourage fathers' attendance at labour and/or birth
Our labour rooms do not offer sufficient privacy to enable fathers to attend labour	
Our delivery rooms do not offer sufficient privacy to enable fathers to attend labour and/or birth	Section Two
The practice of encouraging fathers to attend childbirth in our facility is fully functional	Section Three

**Table 2:** Proposed new items for the MPAC-QMS after round one

*Proposed question/item*	*Section where proposed item may fit*
I understand that a woman may not want her husband/partner present at labour and/or birth	Section One
I ask an expectant woman if she wants the father of the baby present at delivery	Section One

Modifications related to the confusion of using the term ‘father’, confusing wording, irrelevant questions, and leading questions.

### Results of delphi survey round 2

Following round two, nine items of MPAC-QHMUs achieved 80% consensus cut-off but needed some amendments (see Table 3). For the MPAC-QMS, four items achieved 80% agreement cut-off but needed some corrections in their structuring, and four were deleted (see Table 4).

**Table 3:**
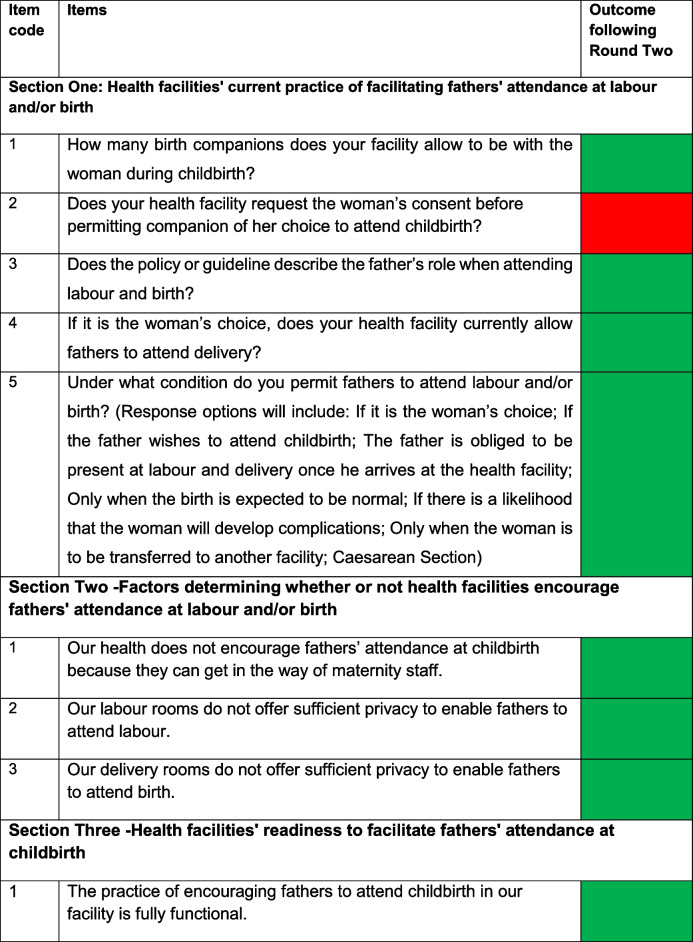
Items of the MPAC-QHMUs sent for round two

Reached 80% and over:

Reached below 80% of agreement cut-off and were deleted:

**Table 4:**
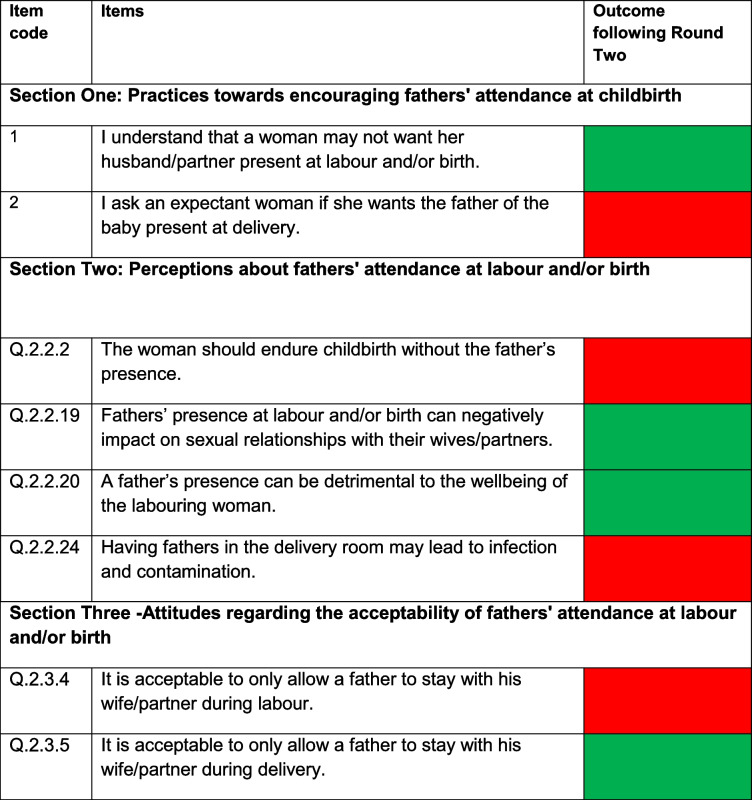
Items of the MPAC-QMS sent for round two

Reached 80% and over:

Reached below 80% of agreement cut-off and were deleted:

After round two, amendments were identified to improve clarity and relevance, minimise bias and avoid overlap. In response to comments, and for consistency and inclusivity, the term ‘father’ was replaced with ‘male partner’. Following round two and these additional revisions, 42 items for the MPAC-QHMUs and 64 items for the MPAC-QMS were retained for round three.

### Results of delphi survey round 3

After round three, one item of the MPAC-QHMUs and three items of the MPAC-QMS were eliminated. Furthermore, seven items were rephrased to address clarity, reduce ambiguity, and correct typographical errors in some of them. After all three rounds, 43 items were retained for the MPAC-QHMUs and 61 items were retained for the MPAC-QMS (see supplementary information 3 and 4 for the final versions of the two questionnaires).

## Discussion

Two questionnaires (the MPAC-QHMUs and the MPAC-QMS) were developed to capture health facilities’ current practice, and service and individual staff level factors which could affect women’s choice to have a male partner attend childbirth. It is proposed that these questionnaires could also be used to identify areas for practice improvement in how health facilities approach the women’s right to have a companion of her choice, including her male partner, during labour and birth. The two questionnaires could also be used to identify both organisational and individual staff attitudes that drive the success and/or the failure of policies aimed at integrating the women’s choices of a birth companion within health facilities [59]. More particularly, the MPAC-QHMUs could serve to inform implementation strategies such as ensuring privacy in the labour wards, policies and protocol development, and training maternity staff and the woman’s chosen companion about birth companionship for health institutions seeking to incorporate male partners’ attendance at childbirth in maternal and neonatal care [60].

The face and content validity of the MPAC-QHMUs and the MPAC-QMS were tested through a three-round modified Delphi survey. The final questionnaire items were judged relevant by experts to explore current practice, service level factors, and maternity staff perceptions and attitudes concerning the acceptability of male partners’ attendance at childbirth. This consensus method was chosen as a rigorous and efficient approach to develop and validate the questionnaires [55]. Unlike other consensus methods such as Nominal Group Technique [61, 62], the modified Delphi approach used to develop and validate the MPAC-QHMUs and the MPAC-QMS was robust in that it combined elements of quantitative measurements with qualitative approaches to assess the content and face validity of the two questionnaires [63].

Through this modified Delphi survey, the content validity and/or relevance of the MPAC-QHMUs and the MPAC-QMS was improved as new items were added such as organisational context and training. Similar to other studies that have applied the Delphi consensus approach to construct questionnaires [64, 65], and other tools [66], the Delphi survey contributed to the reduction of the length of each questionnaire across all three rounds. It was important to produce a final version of each questionnaire which covered all essential relevant components while also minimising the response burden among the target participants [67].

Use of experts to systematically review the content of the two questionnaires led to substantially improved wording of the items, reduction in duplicate items, and elimination of irrelevant items [58]. The panel emphasised the aspect of women’s choices in their feedback. For example, some women might have different preferences for when their male partner could be present at childbirth. Based on these comments, some items were divided into two; an item about labour and another about birth. It was important to have items about attendance at labour and others about birth because some facilities might have different policies for labour and birth regardless of the women’s preferences and consent. The use of the word ‘birth’ raised debate as some panel members suggested the use of the word ‘delivery’. It is probable that different wording suggestions were influenced by the panel members’ professional backgrounds. Some participants were from a midwifery background, and others from an obstetric background and these two disciplines can differ in the way they treat the concept of childbirth and the vocabulary used to describe and discuss concepts around it [68, 69]. The final decision was to use the term ‘birth’ because it empowers the woman and embodies childbearing as a natural occurring event and the woman as the agent of action of giving birth [68, 69].

### Limitations

The use of the expert panel to assess the relevance and clarity of the two questionnaires, despite having been done rigorously and free from any group pressure, the modified version of Delphi methodology cannot guarantee that all flaws in the wording of the items were addressed. In addition, it cannot be ascertained that all relevant aspects of the topic under study were covered. Although our intention was to obtain 100% response rate for all three rounds, round two and three had a panel with fewer than twelve who completed round one because of the unavailability of some panel members. However, this attrition did not impact the consensus obtained for the final versions of MPAC-QHMUs and MPAC-QMS since across all three rounds an average of 80% response rate was achieved.

Another limitation was that the Delphi study to assess the face and content of the MPAC-QHMUs and MPAC-QMS was only developed with experts and did not include the perspectives of consumers.

Whilst we intended to have a panel of experts from geographically diverse LMICs, the countries represented by the panel were predominantly Eastern African. This lack of representatives from all geographical regions may limit the perspectives gained on the survey items’ applicability in wider health system and cultural contexts. The MPAC-QHMUs was meant to capture health systems and organizational factors impacting male partners’ companionship. However, the lack of health systems researchers and lack of health facility administrators on the Delphi panel may not offer comprehensive perspectives on this survey tool from a health services/administrative vantage point.

## Conclusion

Items for the MPAC-QHMUs and MPAC-QMS were robustly generated from the literature and the thorough iterative face and content validation process. To the researcher’s knowledge, the two questionnaires are the first that have been developed to examine current practice and moderators of male partners’ attendance at childbirth in health facilities. Researchers can use the MPAC-QHMUs to determine the prevalence of facilities’ practices regarding implementation of birth companionship, current practices underlying paternal attendance at childbirth, and to identify factors that may facilitate or inhibit the implementation of male partners’ attendance at childbirth in health facilities. The MPAC-QMS could be used to assess maternity staff practices, perceptions, and views about the acceptability and feasibility of male partners’ attendance, which may impact on the successful implementation of any new policies or practices.

The MPAC-QHMUs and the MPAC-QMS are newly-developed. Although the MPAC-QHMUs and MPAC-QMS were piloted in selected health facilities in Rwanda (manuscript under preparation), there is a need for further validation of acceptability and feasibility of administering these questionnaires in different cultural contexts and other languages than English.

## Supplementary Information

**Additional file 1:** **Supplementary information N**^**o**^**: 1.** Experts’ recommendations on items for MPAC-QHMUs sent for assessments in round one.

**Additional file 2:** **Supplementary Information N**^**o**^**: 2.** Experts’ recommendations on items for MPAC-QMS sent for assessments in round one.

**Additional file 3.** QUESTIONNAIRE FOR THE HEADS OF MATERNITY UNITS.

**Additional file 4.** QUESTIONNAIRE FOR MATERNITY STAFF.

## Data Availability

Data from this study can obtained through email by contacting Dr. Thierry Claudien Uhawenimana at tcuhawenimana@gmail.com.

## References

[1] Organization WH. WHO recommendations on intrapartum care for a positive childbirth experience. World Health Organization; 2018. 30070803

[2] Leavitt JW. Make room for daddy: The journey from waiting room to birthing room. Univ of North Carolina Press; 2009.

[3] Linn JG, Wilson DR, Fako TT. Historical Role of the Father: Implications for Childbirth Education. Int J childbirth Educ. 2015;30(1).

[4] King L (2017). Hiding in the Pub to Cutting the Cord? Men’s Presence at Childbirth in Britain c. 1940s–2000s. Soc Hist Med..

[5] Bohren MA, Vogel JP, Hunter EC, Lutsiv O, Makh SK, Souza JP (2015). The mistreatment of women during childbirth in health facilities globally: a mixed-methods systematic review. PLoS Med.

[6] Bryder L (2015). Fathers and Hospital Childbirth in New Zealand. Soc Hist Med.

[7] Premberg A, Lundgren I (2006). Fathers’ experiences of childbirth education. J Perinat Educ.

[8] Balde MD, Nasiri K, Mehrtash H, Soumah AM, Bohren MA, Diallo BA, et al. Labour companionship and women’s experiences of mistreatment during childbirth: results from a multi-country community-based survey. BMJ Glob Heal [Internet]. 2020;5(Suppl. 2). Available from: https://gh.bmj.com/content/5/Suppl_2/e003564 10.1136/bmjgh-2020-003564 PMC7684665 33234502

[9] Destaw A. Assessment of husband involvement during Pregnancy and child birth in AkakiKaliti sub-city. Addis Ababa. 2014;

[10] Ampt F, Mon MM, Than KK, Khin MM, Agius PA, Morgan C (2015). Correlates of male involvement in maternal and newborn health: a cross-sectional study of men in a peri-urban region of Myanmar. BMC Pregnancy Childbirth.

[11] KyiMar W, Shibanuma A, NweNwe O, Fillman TJ, YuMon S, Jimba M (2015). Are husbands involving in their spouses’ utilization of maternal care services?: A cross-sectional study in Yangon, Myanmar. PLoS ONE.

[12] Carter M (2002). Husbands and maternal health matters in rural Guatemala: Wives’ reports on their spouses’ involvement in pregnancy and birth. Soc Sci Med.

[13] Carter MW, Speizer I (2005). Salvadoran fathers’ attendance at prenatal care, delivery, and postpartum care. Rev Panam Salud Publica.

[14] He H, Vehviläinen-Julkunen K, Qian X, Sapountzi-Krepia D, Gong Y, Wang W (2015). Fathers’ feelings related to their partners’ childbirth and views on their presence during labour and childbirth: A descriptive quantitative study. Int J Nurs Pract.

[15] Singh A, Ram F. Men’s involvement during pregnancy and childbirth: evidence from rural Ahmadnagar, India. Popul Rev. 2009;48(1).

[16] Oboro VO, Oyeniran AO, Akinola SE, Isawumi AI (2011). Attitudes of Nigerian women toward the presence of their husband or partner as a support person during labor. Int J Gynecol Obstet.

[17] Diniz CSG, d’Orsi E, Domingues RMSM, Torres JA, Dias MAB, Schneck CA (2014). Implementation of the presence of companions during hospital admission for childbirth: data from the Birth in Brazil national survey. Cad Saude Publica.

[18] Aarons GA, Sommerfeld DH, Walrath-Greene CM (2009). Evidence-based practice implementation: the impact of public versus private sector organization type on organizational support, provider attitudes, and adoption of evidence-based practice. Implement Sci.

[19] Weng Y-H, Kuo KN, Yang C-Y, Lo H-L, Chen C, Chiu Y-W (2013). Implementation of evidence-based practice across medical, nursing, pharmacological and allied healthcare professionals: a questionnaire survey in nationwide hospital settings. Implement Sci.

[20] Abushaikha L, Massah R (2013). Perceptions of barriers to paternal presence and contribution during childbirth: an exploratory study from Syria. Birth.

[21] Bohren MA, Berger BO, Munthe-Kaas H, Tunçalp Ö (2019). Perceptions and experiences of labour companionship: a qualitative evidence synthesis. Cochrane Database Syst Rev.

[22] Carter M (2002). Husbands and maternal health matters in rural Guatemala: wives’ reports on their spouses’ involvement in pregnancy and birth. Soc Sci Med.

[23] Dumbaugh M, Tawiah-Agyemang C, Manu A, ten Asbroek GHA, Kirkwood B, Hill Z (2014). Perceptions of, attitudes towards and barriers to male involvement in newborn care in rural Ghana, West Africa: a qualitative analysis. BMC Pregnancy Childbirth.

[24] Kaye DK, Kakaire O, Nakimuli A, Osinde MO, Mbalinda SN, Kakande N (2014). Male involvement during pregnancy and childbirth: men’s perceptions, practices and experiences during the care for women who developed childbirth complications in Mulago Hospital Uganda. BMC Pregnancy Childbirth.

[25] Kululanga LI, Sundby J, Chirwa E, Malata A, Maluwa A (2012). Barriers to husbands’ involvement in maternal health care in a rural setting in Malawi: a qualitative study. J Res Nurs Midwifery.

[26] Emelonye AU, Pitkäaho T, Aregbesola A, Vehviläinen-Julkunen K (2017). Barriers to spousal contribution to childbirth pain relief in Nigeria. Int Nurs Rev.

[27] Kwambai TK, Dellicour S, Desai M, Ameh CA, Person B, Achieng F (2013). Perspectives of men on antenatal and delivery care service utilisation in rural western Kenya: a qualitative study. BMC Pregnancy Childbirth.

[28] Nesane K, Maputle SM, Shilubane H (2016). Male partners’ views of involvement in maternal healthcare services at Makhado Municipality clinics, Limpopo Province, South Africa. African J Prim Heal Care Fam Med.

[29] Mullany BC (2006). Barriers to and attitudes towards promoting husbands’ involvement in maternal health in Katmandu. Nepal Soc Sci Med.

[30] Mukamurigo J, Dencker A, Ntaganira J, Berg M (2017). The meaning of a poor childbirth experience–a qualitative phenomenological study with women in Rwanda. PLoS ONE.

[31] Secka E (2010). Men’s involvement in care and support during pregnancy and childbirth.

[32] Sengane MLM, Nolte AGW (2011). The expectations of fathers concerning care provided by midwives to the mothers during labour. Heal SA Gesondheid.

[33] Souza SRRK, Gualda DMR. The experience of women and their coaches with childbirth in a public maternity hospital. Texto Context. 2016;25.

[34] Påfs J, Rulisa S, Musafili A, Essén B, Binder-Finnema P (2016). ‘You try to play a role in her pregnancy’-a qualitative study on recent fathers’ perspectives about childbearing and encounter with the maternal health system in Kigali, Rwanda. Glob Health Action.

[35] Hollins MC (2008). A tool to measure fathers’ attitudes and needs in relation to birth. Br J Midwifery.

[36] Premberg Å, Taft C, Hellström A-L, Berg M (2012). Father for the first time - development and validation of a questionnaire to assess fathers’ experiences of first childbirth (FTFQ). BMC Pregnancy Childbirth.

[37] Daniels E, Arden-Close E, Mayers A (2020). Be quiet and man up: a qualitative questionnaire study into fathers who witnessed their Partner’s birth trauma. BMC Pregnancy Childbirth.

[38] Sapountzi-Krepia D, Lavdaniti M, Dimitriadou A, Psychogiou M, Sgantzos M, He H-G (2010). Fathers’ Feelings and Experience Related to their Wife/Partner’s Delivery in Northern Greece. Open Nurs J.

[39] Webb R, Smith AM, Ayers S, Wright DB, Thornton A (2021). Development and Validation of a Measure of Birth-Related PTSD for Fathers and Birth Partners: The City Birth Trauma Scale (Partner Version). Front Psychol.

[40] Bukkavesa S (1982). Nursing students’ perceptions about fathers’ presence in the delivery room.

[41] Uhawenimana TC. Exploration of factors affecting male partner’s attendance at childbirth in Rwandan health facilities. University of Dundee; 2021.

[42] Wiltsey Stirman S, Kimberly J, Cook N, Calloway A, Castro F, Charns M (2012). The sustainability of new programs and innovations: a review of the empirical literature and recommendations for future research. Implement Sci.

[43] Rattray J, Jones MC (2007). Essential elements of questionnaire design and development. J Clin Nurs.

[44] Colton D, Covert RW. Designing and constructing instruments for social research and evaluation. Wiley. 2007.

[45] Aday LA, Cornelius LJ. Designing and conducting health surveys: a comprehensive guide. Wiley. 2006.

[46] Streiner DL, Norman GR, Cairney J (2015). Health measurement scales: a practical guide to their development and use.

[47] Oosterveld P, Vorst H, Smits N (2019). Methods for questionnaire design: a taxonomy linking procedures to test goals. Qual Life Res.

[48] Putnam SP, Helbig AL, Gartstein MA, Rothbart MK, Leerkes E (2014). Development and assessment of short and very short forms of the Infant Behavior Questionnaire-Revised. J Pers Assess.

[49] Taherdoost H. Validity and reliability of the research instrument; how to test the validation of a questionnaire/survey in a research. How to test Valid a Quest a Res (August 10, 2016). 2016;

[50] Landeta J (2006). Current validity of the Delphi method in social sciences. Technol Forecast Soc Change.

[51] Keeney S, McKenna H, Hasson F. The Delphi technique in nursing and health research. Wiley. 2011.

[52] Shariff N (2015). Utilizing the Delphi survey approach: A review. J Nurs Care.

[53] McPherson S, Reese C, Wendler MC (2018). Methodology update: Delphi studies. Nurs Res.

[54] Bolarinwa OA (2015). Principles and methods of validity and reliability testing of questionnaires used in social and health science researches. Niger Postgrad Med J.

[55] Jorm AF (2015). Using the Delphi expert consensus method in mental health research. Aust New Zeal J Psychiatry.

[56] Vogel C, Zwolinsky S, Griffiths C, Hobbs M, Henderson E, Wilkins E (2019). A Delphi study to build consensus on the definition and use of big data in obesity research. Int J Obes.

[57] Fauconnier A, Staraci S, Daraï E, Descamps P, Nisolle M, Panel P (2018). A self-administered questionnaire to measure the painful symptoms of endometriosis: results of a modified DELPHI survey of patients and physicians. J Gynecol Obstet Hum Reprod..

[58] Polit DF, Beck CT. Nursing research: Generating and assessing evidence for nursing practice Tenth ed. New York, Baltimore, Philadelphia: Lippincott William & Wilkins; 2017.

[59] Kabakian-Khasholian T, Portela A (2017). Companion of choice at birth: factors affecting implementation. BMC Pregnancy Childbirth.

[60] Bharti J, Kumari A, Zangmo R, Mathew S, Kumar S, Sharma AK (2021). Establishing the practice of birth companion in labour ward of a tertiary care centre in India—a quality improvement initiative. BMJ Open Qual..

[61] James D, Warren-Forward H (2015). Research methods for formal consensus development. Nurse Res.

[62] Harvey N, Holmes CA (2012). Nominal group technique: an effective method for obtaining group consensus. Int J Nurs Pract.

[63] Fink-Hafner D, Dagen T, Doušak M, Novak M, Hafner-Fink M (2019). Delphi method: strengths and weaknesses. Adv Methodol Stat.

[64] Delgado A, de Oliveira PdNF, de Góes PSA, Lemos A (2019). Development and analysis of measurement properties of the “maternal perception of childbirth fatigue questionnaire”(MCFQ). Brazilian J Phys Ther..

[65] Gagnon AJ, DeBruyn R, Essén B, Gissler M, Heaman M, Jeambey Z (2014). Development of the Migrant Friendly Maternity Care Questionnaire (MFMCQ) for migrants to Western societies: an international Delphi consensus process. BMC Pregnancy Childbirth.

[66] Sauvegrain P, Chantry AA, Chiesa-Dubruille C, Keita H, Goffinet F, Deneux-Tharaux C (2019). Monitoring quality of obstetric care from hospital discharge databases: A Delphi survey to propose a new set of indicators based on maternal health outcomes. PLoS ONE.

[67] Rolstad S, Adler J, Rydén A (2011). Response burden and questionnaire length: is shorter better? A review and meta-analysis. Value Heal.

[68] Hunter LP (2006). Women give birth and pizzas are delivered: language and western childbirth paradigms. J Midwifery Womens Health.

[69] Sanders L. Empowering women through the birth process: midwifery vs. The medical model. 2013.

