# Supplementary material for: Development and early validation of questionnaires to assess system level factors affecting male partners’ attendance at childbirth in LMICs
Source: BMC Pregnancy Childbirth. 2023 Apr 17;23:258. doi: 10.1186/s12884-023-05580-y (PMC10108494; doi:10.1186/s12884-023-05580-y)
Supplement: Supplementary file 1 — Additional file 1: Supplementary information No: 1. Experts’ recommendations on items for MPAC-QHMUs sent for assessments in round one. [file 12884_2023_5580_MOESM1_ESM.docx]

**Supplementary information N^o^: 1: Experts’ recommendations on items for MPAC-QHMUs sent for assessments in round one**

| **Item code** | **Items** | **Outcome following Round One** |
| --- | --- | --- |
| **Section One: Health facilities' current practice of facilitating fathers' attendance at labour and/or birth** | | |
| Q1.1.1 | Does your health facility allow expectant women to have birth companions of their choice during childbirth? |  |
| Q1.1.2 | Who does your health facility allow to stay with the woman throughout childbirth? |  |
| Q1.1.3 | Does your health facility have a policy or a guideline on birth companions? |  |
| Q1.1.4 | If there is a policy or guideline, does your health facility have a copy of the policy/guideline? |  |
| Q1.1.5 | Does the policy or guideline address fathers’ attendance at labour and/or birth? |  |
| Q1.1.6 | If it is the woman’s choice, does your health facility currently allow fathers to attend labour and/or birth if it is the woman’s choice? |  |
| Q1.1.7 | If the woman wants the father to be present, does your health facility inform the couple about the period when the father is allowed to stay in the labour room? |  |
| Q1.1.8 | When does your health facility permit fathers to attend labour and/or birth? |  |
| Q1.1.9 | If the father is allowed to stay with the mother during labour, does your health facility provide him with information about labour support? |  |
| Q1.1.10 | Is the father asked to leave the room for routine procedures during labour? |  |
| Q1.1.11 | If the woman wants the father of the baby to be present at birth, does your health facility inform the father on what to expect during his stay? |  |
| Q1.1.12 | If the father is allowed to stay with the mother during birth, does your health facility provide the father with appropriate clothing for example gown and shoes before entering the delivery ward? |  |
| Q1.1.13 | Does your health facility have any preparatory classes for fathers about labour and birth support? |  |
| Q1.1.14 | Does your health facility have any form of recording about how many fathers attend labour and/or birth? |  |
| **Section Two: Factors determining whether or not health facilities encourage fathers' attendance at labour and/or birth.** | | |
| Q1.2.1 | Availability of a national policy about birth companionship will guide our health facility to encourage fathers’ attendance at childbirth. |  |
| Q1.2.2 | Our institutional policies don’t support fathers’ attendance at childbirth. |  |
| Q1.2.3 | Maternity staff at this facility are not aware of the woman’s right to have a birth companion of her choice. |  |
| Q1.2.4 | The senior management of our health facility is not willing to encourage fathers’ presence at labour and/or birth. |  |
| Q1.2.5 | Fathers can evaluate maternity staff actions during labour and/or birth. |  |
| Q1.2.6 | Limited space in the labour and delivery rooms limits our health facility’s ability to encourage fathers’ attendance at labour and/or birth. |  |
| Q1.2.7 | Our labour rooms do not offer sufficient privacy to enable fathers to attend labour and/or birth. |  |
| Q1.2.8 | The high number of births conducted by our maternity staff does not facilitate them to encourage fathers to attend labour and/or birth. |  |
| Q1.2.9 | Maternity staff in our health facility are not willing to permit fathers to be present at labour and/or birth. |  |
| Q1.2.10 | Lack of individual labour rooms in our health facility affect fathers’ presence at labour and/or birth. |  |
| Q1.2.11 | Our health facility fears that allowing fathers to attend labour and/or birth may spread infection to the woman and the baby. |  |
| Q1.2.12 | Maternity staff in this facility have limited training on facilitating fathers’ attendance at childbirth. |  |
| Q1.2.13 | Our facility lacks amenities such as toilets and beds for fathers who may wish to stay overnight with their wives/partners. |  |
| Q1.2.14 | Our facility does not encourage fathers’ presence at labour and birth because they may become aggressive. |  |
| Q1.2.15 | Our health facility does not encourage fathers’ attendance at childbirth because of fear of litigation. |  |
| Q1.2.16 | Our health facility does not encourage fathers’ attendance at childbirth because our focus of care is the mother and the baby. |  |
| Q1.2.17 | Fathers’ presence at childbirth distracts maternity staff from attending to the mother during childbirth. |  |
| Q1.2.18 | Our health facility does not encourage fathers’ attendance at childbirth because we consider birth companionship to be a female role. |  |
| Q1.2.19 | Our health facility does not encourage fathers to attend childbirth because men are not culturally expected to be present at childbirth. |  |
| **Section Three -Health facilities' readiness to facilitate fathers' attendance at childbirth** | | |
| Q1.3.1 | Our health facility is considering encouraging fathers to attend labour/or birth. |  |
| Q1.3.2 | Our health facility has made good efforts to implement fathers’ attendance at childbirth but we still have some work to do. |  |
| Q1.3.3 | Our health facility has initiated fathers’ attendance at childbirth, and it is fully functional. |  |
| Q1.3.4 | We believe that allowing fathers to attend childbirth will change men’s perceptions of childbirth. |  |
| Q1.3.5 | We believe that allowing fathers’ attendance at childbirth in our health facility will strengthen our mission to promote respectful maternity care. |  |
| Q1.3.6 | We believe that implementing fathers’ attendance at childbirth will enable our health facility to involve men in other safe motherhood programmes. |  |
| Q1.3.7 | There is a high demand for paternal involvement in childbirth from couples attending this facility. |  |
| Q1.3.8 | Allowing fathers’ attendance at childbirth is one of our facility’s core values. |  |
| Q1.3.9 | Introducing fathers to attend childbirth is not in line with our health facility’s principles. |  |
| Q1.3.10 | Introducing fathers’ attendance at childbirth will require our facility to organise specific antennal classes for them. |  |
| Q1.3.11 | Our health facility is ready to put in place guidelines to facilitate fathers’ attendance at childbirth. |  |
| Q.1.3.12 | Our health facility is ready to put in place all necessary resources to enable fathers’ presence at labour and birth. |  |
| Q1.3.13 | Our health facility is ready to put in place all necessary resources to enable fathers’ presence at labour and birth |  |
| Q1.3.14 | Managers of our health facility are committed to sustaining fathers’ attendance at childbirth. |  |

***Reached 80% and over:***

***Reached 80% cut-off agreement but needed revisions:***

***Garnered between 60 and 79% and were sent for round two Delphi:***

***Reached below 60% of agreement cut-off and were deleted:***

**List of items of the MPAC-QHMUs that were deleted and reasons for their deletion as recommended by Delphi panel**

| ***Code*** | ***Item*** | *% Rating* 3 (relevant) or 4 (very relevant) | ***Comments provided by the panel members*** | ***Justification for deleting the item*** |
| --- | --- | --- | --- | --- |
| **Section Two -Factors determining whether or not health facilities encourage fathers' attendance at labour and/or birth** | | | | |
| Q1.2.10 | Lack of individual labour rooms in our health facility affect fathers’ presence at labour and/or birth | 83.3% | -Maybe. Though I think you’d have captured the answer in the question on infrastructure.  -How is this question different from 1.2.6? | The item is repeated elsewhere. |
| Q1.2.4 | The senior management of our health facility is not willing to encourage fathers’ presence at labour and/or birth. | ***100%*** | I am not sure how honest would the unit heads be answering to this question. | Heads of maternity units may not be in a position to answer this item. In addition, the item may invoke biased response. |
| **Section Three -Health facilities' readiness to facilitate fathers' attendance at childbirth** | | | | |
| Q1.3.2 | Our health facility has made good efforts to implement fathers’ attendance at childbirth, but we still have some work to do. | 91.7% | -Hard to measure good efforts. Define an indicator and set a target then measure against that target annually. Based on this comment, this item would not stand for the current project which is explorative in nature. | The item is ambiguous. |
| Q1.3.5 | We believe that allowing fathers’ attendance at childbirth in our health facility will strengthen our mission to promote respectful maternity care. | 91.7% | -I’m not sure it is the best way to promote respectful care. The women are best placed to report on this, otherwise we disempower them if they have to depend on a man’s presence to receive respectful care. | The item not appropriate to be asked the heads of maternity units. |
| Q1.3.9 | Introducing fathers to attend childbirth is not in line with our health facility’s principles. | 91.7% | -It is a repetition of 1.3.8 | Similar to 1.3.8 |
| Q1.3.13 | Our health facility has a space in the maternity ward where fathers talk to other fathers about childbirth topics. | 91.7% | -This statement could be not relevant because, in the Rwandan context, it can be difficult to make this applicable. | Item not relevant to the Rwandan context. |
