# Supplementary material for: Development and early validation of questionnaires to assess system level factors affecting male partners’ attendance at childbirth in LMICs
Source: BMC Pregnancy Childbirth. 2023 Apr 17;23:258. doi: 10.1186/s12884-023-05580-y (PMC10108494; doi:10.1186/s12884-023-05580-y)
Supplement: Supplementary file 2 — Additional file 2: Supplementary Information No: 2. Experts’ recommendations on items for MPAC-QMS sent for assessments in round one. [file 12884_2023_5580_MOESM2_ESM.docx]

**Supplementary Information N^o^: 2: Experts’ recommendations on items for MPAC-QMS sent for assessments in round one**

| **Item code** | **Items** | **Outcome following Round One** |
| --- | --- | --- |
| **Section One: Practices towards encouraging fathers' attendance at childbirth** | | |
| Q2.1.1 | I develop a birth plan with the couple indicating the level of the father’s participation during childbirth. |  |
| Q2.1.2 | I ask an expectant woman if she wants to have the father of the baby present at labour and/or birth. |  |
| Q2.1.3 | I give information to fathers on what to expect during childbirth when they attend antenatal appointments. |  |
| Q2.1.4 | I educate fathers about labour support when they attend antenatal appointments. |  |
| Q2.1.5 | I ask an expectant woman if she wishes the baby’s father to attend labour and/or birth. |  |
| Q2.1.6 | I ask an expectant father if he wishes to attend labour and/or birth. |  |
| Q2.1.7 | I insist that any father who accompanies the woman has to stay with her during labour and birth. |  |
| Q2.1.8 | I teach an expectant father how to support the woman to adopt comfortable positions during labour. |  |
| Q2.1.9 | I teach an expectant father how to provide emotional care for the labouring woman. |  |
| Q2.1.10 | I teach an expectant father how to massage the woman during labour. |  |
| Q2.1.11 | I seek the woman’s consent before informing the father about the care provided to his partner during labour and birth. |  |
| Q2.1.12 | I communicate to fathers about labour and birth progress. |  |
| Q2.1.13 | I involve both parents in decisions about childbirth. |  |
| Q2.1.14 | I listen to the father’s concerns about the woman’s condition during childbirth. |  |
| Q2.1.15 | If a father becomes distressed, I ask him to leave the labour room. |  |
| Q2.1.16 | I ask the father if he wants to watch his baby being born. |  |
| Q2.1.17 | I brief the father with what to expect during delivery. |  |
| Q2.1.18 | I offer the father an opportunity to cut the umbilical cord. |  |
| **Section Two -Perceptions about fathers' attendance at labour** | | |
| Q2.2.1 | Fathers’ presence at labour and birth is one of the ways to make childbirth care more family centred. |  |
| Q2.2.2 | The woman should endure childbirth without the father’s presence. |  |
| Q2.2.3 | The father must not be allowed inside the maternity ward if the mother has a female companion. |  |
| Q2.2.4 | Fathers should be allowed to attend childbirth to witness women’s endurance during childbirth. |  |
| Q2.2.5 | Allowing fathers to attend childbirth protects healthcare providers against any allegations of negligence. |  |
| Q2.2.6 | The father may assure the safety of the mother and the baby when he is allowed to be present at labour and birth. |  |
| Q2.2.7 | Fathers provide verbal reassurance to the mother during labour. |  |
| Q2.2.8 | A father’s presence makes the mother feel less stressed during childbirth. |  |
| Q2.2.9 | A father’s presence make the mother feel more stressed during labour and birth. |  |
| Q2.2.10 | A father’s attendance at birth fosters his bonding with the baby. |  |
| Q2.2.11 | Fathers’ presence at labour and birth relieves the mother’s anxieties and stress. |  |
| Q2.2.12 | A father’s presence at labour and birth increases the mother’s anxieties and stress. |  |
| Q2.2.13 | Fathers need to attend childbirth to develop a better appreciation of the woman. |  |
| Q2.2.14 | The father may assist the mother to do breathing and relaxation exercises. |  |
| Q2.2.15 | Fathers’ presence may impact on couples’ subsequent pregnancy planning. |  |
| Q2.2.16 | The father’s presence can make the woman uncomfortable during labour. |  |
| Q2.2.17 | Fathers’ presence may cause women to desire more attention and sympathy from the maternity staff. |  |
| Q2.2.18 | The father may not wish to help the woman if she needs to go to bathroom. |  |
| Q2.2.19 | Fathers’ presence at labour and/or birth can negatively impact on sexual relationships with their wives/partners. |  |
| Q2.2.20 | A father’s presence can be detrimental to the wellbeing of the labouring woman. |  |
| Q2.2.21 | Fathers’ presence may provoke undesirable emotional reactions from the woman during childbirth. |  |
| Q2.2.22 | The father may faint upon seeing blood during the delivery of the baby. |  |
| Q2.2.23 | Permitting fathers to be present at childbirth can lead to congestion in the maternity ward. |  |
| Q2.2.24 | Having fathers in the delivery room may lead to infection and contamination. |  |
| Q2.2.25 | Fathers’ presence at childbirth will increase stress on healthcare providers. |  |
| Q2.2.26 | Maternity staff may feel uncomfortable when they are watched by fathers while offering care to the mother. |  |
| Q2.2.27 | Fathers get in the way of providers during their care provision to the woman. |  |
| Q2.2.28 | Fathers’ presence does not help at all in the care of the woman during childbirth. |  |
| **Section Three -Attitudes regarding the acceptability of fathers' attendance at labour and/or birth** | | |
| Q.2.3.1 | It is acceptable to allow a father to be present at labour and birth if it is the woman’s choice. |  |
| Q.2.3.2 | It is acceptable for the father to accompany the woman to the hospital and let a female birth companion stay with the woman during childbirth. |  |
| Q.2.3.3 | It is acceptable to oblige fathers to stay with their wives/partners throughout childbirth once they arrive at the place of delivery. |  |
| Q.2.3.4 | It is acceptable to only allow fathers to stay with his wife/partner during labour. |  |
| Q.2.3.5 | It is acceptable to only allow fathers to stay with his wife/partner during delivery. |  |
| Q.2.3.6 | It is acceptable to allow fathers in the maternity ward only when they come to pay the maternity bills. |  |
| Q.2.3.7 | It is acceptable to only allow the father to stay with his wife/partner if she is expected to deliver normally. |  |
| Q.2.3.8 | It is acceptable to allow all fathers to attend childbirth without imposing any limitation on them. |  |
| Q.2.3.9 | It is acceptable for the maternity staff to facilitate fathers to attend childbirth to share the birth experience with their wives/partners. |  |
| Q.2.3.10 | It is acceptable to allow fathers to stay with their wives/partners to offer practical support to them. |  |
| Q.2.3.11 | It is acceptable for healthcare providers to allow the father to take pictures and videos throughout childbirth. |  |
| Q.2.3.12 | It is acceptable for healthcare providers to assist the father to support his wife/partner during labour and birth. |  |
| Q.2.3.13 | It is acceptable to allow fathers to attend Caesarean section. |  |
| Q.2.3.14 | It is acceptable for health providers to engage fathers in providing support to their partners during labour. |  |
| Q.2.3.15 | It is acceptable for health providers to establish a good relationship with the father to enable him to cope with their woman’s labour endurance. |  |
| Q.2.3.16 | It is acceptable to not allow fathers who look anxious to stay with the woman during labour and/or birth. |  |
| Q.2.3.17 | It is acceptable for healthcare providers to support fathers to provide emotional support to their partners during labour and/or birth. |  |
| Q.2.3.18 | It is acceptable for healthcare providers to guide the father to provide massage to the mother during labour. |  |
| Q.2.3.19 | It is acceptable for healthcare providers to provide the father with all the information about the mother’s childbirth progress. |  |
| **Section Four -Attitudes regarding the feasibility of fathers' attendance at labour and/or birth** | | |
| Q.2.4.1 | It is feasible for me to observe hygiene and infection control mechanisms for birth companions including fathers. |  |
| Q.2.4.2 | It is feasible for me to advise fathers about coping mechanism to adopt during labour and birth. |  |
| Q.2.4.3 | It is feasible for me to inform the woman of her right to choose her husband/partner as her birth companion. |  |
| Q.2.4.4 | It is feasible for me to ask the father if he wishes to attend childbirth. |  |
| Q.2.4.5 | It is feasible for me to inform fathers what happens during labour and birth when they attend antenatal appointments. |  |
| Q.2.4.6 | It is feasible for me to permit the father to be with his wife/partner regardless of the type of birth. |  |
| Q.2.4.7 | It is feasible for me to teach fathers how to support the mother to adopt comfortable positions during labour. |  |
| Q.2.4.8 | It is feasible for me show fathers how to massage their partners during labour. |  |
| Q.2.4.9 | It is feasible for me to inform fathers about the progress of labour. |  |
| Q.2.4.10 | It is feasible for me to inform the father about any major decision regarding the care of his wife/partner. |  |
| Q.2.4.11 | It is feasible for me to help fathers articulate their needs during their stay in the maternity ward. |  |
| Q.2.4.12 | It is feasible for me to ask a father to leave the labour room if he becomes distressed. |  |
| Q.2.4.13 | It is feasible for me to listen to the father’s concerns about the woman’s condition during childbirth. |  |
| Q.2.4.14 | It is feasible for me to show the father where to sit while staying in the labour ward. |  |
| Q.2.4.15 | It is feasible for me to welcome fathers in the delivery room to welcome their baby. |  |
| Q.2.4.16 | It is feasible for me to advise fathers about appropriate clothing to wear before entering the delivery room. |  |
| Q.2.4.17 | It is feasible for me to thank the father for his efforts to be with his wife/partner during labour and birth. |  |

***Reached 80% and over:***

***Reached 80% cut-off agreement but needed revisions:***

***Garnered between 60 and 79% and were sent for round two Delphi:***

***Reached below 60% of agreement cut-off and were deleted:***

**List of items of the MPAC-QMS that were deleted and reasons for their deletion as recommended by Delphi panel**

| ***Code*** | ***Item*** | ***% rating 3 (quite relevant) and 4(very relevant) combined*** | ***Suggestions for improvement as per recommended by expert panel members*** | ***Decision made by the researcher and justification*** |
| --- | --- | --- | --- | --- |
| **Maternity staff: Section One -Practices towards encouraging fathers' attendance at childbirth** | | | | |
| Q2.1.17 | I brief the father with what to expect during delivery. | 91.7% | Similar to 2.1.3 | Similar to 2.1.3 |
| **Section Two -Perceptions about fathers' attendance at labour and/or birth** | | | | |
| Q2.2.6 | The father may assure the safety of the mother and the baby when he is allowed to be present at labour and birth. | 83.3% | -It implies the health workers are not to be trusted. Yet they are professional. | The item may be sensitive to some health professionals. |
| Q2.2.11 | Fathers’ presence at labour and birth relieves the mother’s anxieties and stress. | 83.3% | Similar to 2.2.9 | Similar to 2.2.9 |
| Q2.2.12 | A father’s presence at labour and birth increases the mother’s anxieties and stress. | 91.7% | Similar to 2.2.9 | Similar to 2.2.9 |
| Q2.2.17 | Fathers’ presence may cause women to desire more attention and sympathy from the maternity staff. | 58.3% |  | The item is below 60% agreement cut-off. |
| Q2.2.18 | The father may not wish to help the woman if she needs to go to bathroom. | 58.3% |  | The item is below 60% agreement cut-off. |
| **Section Three -Attitudes regarding the acceptability of fathers' attendance at labour and/or birth** | | | | |
| Q2.3.6 | It is acceptable to allow fathers in the maternity ward only when they come to pay the maternity bills. | 75% | -Important to ask!  -Are the bills paid at the maternity ward? I’d think bills are paid to a cashier sitting in a finance office just outside of where there is a labour action. | Based on the maternity ward set up, this item is not relevant. |
