# Supplementary material for: Development and early validation of questionnaires to assess system level factors affecting male partners’ attendance at childbirth in LMICs
Source: BMC Pregnancy Childbirth. 2023 Apr 17;23:258. doi: 10.1186/s12884-023-05580-y (PMC10108494; doi:10.1186/s12884-023-05580-y)
Supplement: Supplementary file 3 — Additional file 3. QUESTIONNAIRE FOR THE HEADS OF MATERNITY UNITS. [file 12884_2023_5580_MOESM3_ESM.docx]

QUESTIONNAIRE FOR THE HEADS OF MATERNITY UNITS

# Page 1: ABOUT THIS STUDY

Participant Number (Leave the space blank, to be completed by the data collection team)


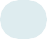


*1.*


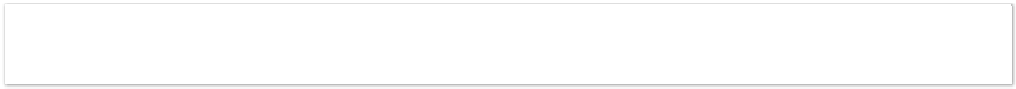


....../....../2019

*1.a.*


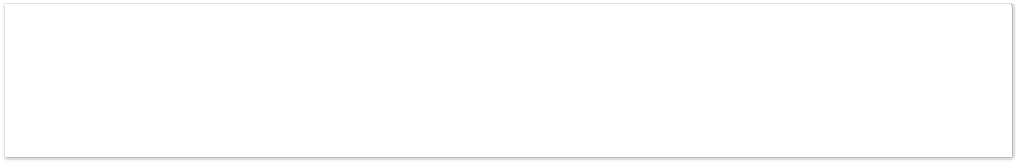

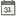


Dates need to be in the format 'DD/MM/YYYY', for example 27/03/1980.

(dd/mm/yyyy)

### Male partners’ attendance at childbirth in Rwandan health facilities: current practice, service level factors, and health providers’ views.

Dear Participant,

You are invited to participate in our study titled 'Male partners' attendance at childbirth in Rwandan health facilities: current practice, service level factors, and health providers' views'.

As you will have read in the Participant Information Sheet provided to you, your participation in this study will contribute to obtaining preliminary data on perceptions and attitudes regarding the acceptability and feasibility of male partners’ attendance at childbirth in Rwandan health facilities. Your participation will provide data about the extent to which maternity staff in Rwandan health facilities facilitate male partners’ attendance at labour and/or birth. It is expected that information from this study may inform policy on male partners’ attendance at childbirth if it is the woman’s choice. It may also inform interventions targeting barriers to women’s choice of birth companion.

Once again, we thank you for taking time to complete this questionnaire.

### Consent to participate in the study

You are not required to sign a separate consent form. You will confirm your voluntary participation in the study by ticking **Yes** or **No** below. Once you agree to participate in the study, the return of completed questionnaires will be taken as consent.

I am interested to participate in this study.


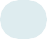


*2.*


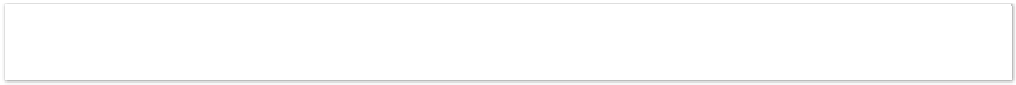

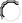

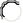


Yes No

If you opted to participate in the study, do you feel comfortable to read and answer all questions in this questionnaire in English language?


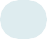


*3.*


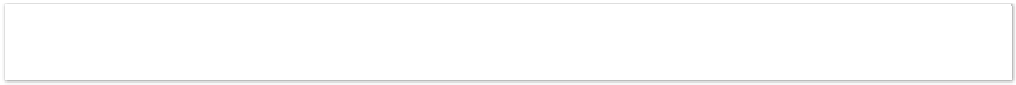

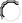

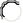


Yes No

If the answer is no, do not continue to the next stage of this survey. Thank you for taking your time to participate in our study.

*This questionnaire comprises four sections:*

*In section 1, you are asked to provide some background information of your health facility.*

## Section 2 asks you about the current practice of male partners' attendance at childbirth in their health facilities.

*Section 3 comprises items about factors determining whether or not health facilities facilitate male partners to attend labour and/or birth.*

## Section 4 comprises items about health facilities' readiness to facilitate male partners' attendance at childbirth.

Section 1 asks you about some contextual information about your health facility.


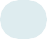


*4.*

|  | Public | Private |
| --- | --- | --- |
| 1. Please tick from the two types of health facilities which one corresponds to your health facility. |  |  |

*4.a.*

|  | Health Centre | District Hospital | Referral Hospital | Private (clinic, polyclinic or hospital) |
| --- | --- | --- | --- | --- |
| 2. Your facility is registered in the Ministry of Health as a: |  |  |  |  |

*4.a.i.*

|  | General medical doctor (s) | Obstetrician (s) | Registered Midwife (ves) | Registered Nurse (s) | Anaesthetist (s) | Paediatrician(s) |
| --- | --- | --- | --- | --- | --- | --- |
| 3. Number of staff working in the maternity unit par each set of professional. |  |  |  |  |  |  |

*4.a.i.a.*

|  | Number of beds in the labour room | Number of beds in the delivery room |
| --- | --- | --- |
| 4. What is the bed capacity of your maternity ward? (Please report the number of the bed capacity in the labour and delivery wards) |  |  |

*4.a.i.a.i.*

|  | Number of births |
| --- | --- |
| 5. On average, how many births are conducted here per day? |  |

# Page 2: Section 2 - Health facilities' current practice of facilitating male partners' attendance at labour and/or birth

In this section, you are asked to report your health facility's current practice regarding birth companions, particularly male partners' attendance at childbirth. The first seven questions (Q1.1.1 to 1.1.7) are about birth companions in general. The next nine questions ask you to report about your healt facility's practice of male partners' attendance at labour and/or birth (Q1.1.8 up to 1.1.16).


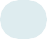


*5.*

|  | Yes | No |
| --- | --- | --- |
| Q1.1.1. Does your health facility allow women to have birth companions of their choice during childbirth? |  |  |
| Q1.1.2. Does your health facility request the woman’s consent before permitting a companion of her choice to attend childbirth? |  |  |

*5.a.*

|  | Number of companions allowed |
| --- | --- |
| Q1.1.3. How many birth companions does your facility allow to be with the woman during childbirth? |  |

*5.a.i.*

|  | Husband/partner | Female relative (s) | Female friend | Any other person the woman chooses (please specify):  ……. |
| --- | --- | --- | --- | --- |
| Q1.1.4. Who does your health facility allow to stay with the woman throughout childbirth? |  |  |  |  |

*5.a.i.a.*

|  | Yes | No |
| --- | --- | --- |

| Q1.1.5. Does your health facility have any document such as a policy or protocol on birth companions? |  |  |
| --- | --- | --- |
| Q1.1.6. If the document is available, does the maternity service have a copy of it? |  |  |
| Q1.1.7. Does this document provide clear guidance on male partners' attendance at childbirth? |  |  |

*5.a.i.a.i.*

|  | Yes | No |
| --- | --- | --- |
| Q1.1.8. If it is the woman’s choice, does your health facility currently allow male partners to attend labour? |  |  |
| Q1.1.9. If it is the woman’s choice, does your health facility currently allow male partners to attend the delivery of the baby? |  |  |

If the answer to **Q1.1.8** and **Q1.1.9** is no, please go to section two (1.2.1).

*5.a.i.a.i.a.*

|  | Only during visiting hours | During labour only | During birth only | During labour and birth | Other (please specify):.................. |
| --- | --- | --- | --- | --- | --- |
| Q1.1.10. When does your health facility permit male partners to enter the maternity ward to be with the woman? |  |  |  |  |  |

*5.a.i.a.i.a.i.*

|  | The | Only when the birth is expected to be normal | If there is a likelihood that the woman will develop complications | Only when the woman is to be transferred to another facility |  |  |
| --- | --- | --- | --- | --- | --- | --- |
|  | male |  |  |  |  |  |
|  | partner |  |  |  |  |  |
|  | is |  |  |  |  |  |
|  | obliged |  |  |  |  |  |
|  | to be |  |  |  |  |  |
|  | present |  |  |  |  | Other, |
|  | at |  |  |  | Caesarean | please |
|  | labour |  |  |  | Section | specify:....... |
|  | and |  |  |  |  |  |
|  | delivery |  |  |  |  |  |
|  | once he |  |  |  |  |  |
|  | arrives |  |  |  |  |  |
|  | at the |  |  |  |  |  |
|  | health |  |  |  |  |  |
|  | facility |  |  |  |  |  |
| Q1.1.11.  Under what condition do you permit male partners to attend childbirth? |  |  |  |  |  |  |

*5.a.i.a.i.a.i.a.*

|  | Yes | No |
| --- | --- | --- |
| Q1.1.12. When the male partner is allowed to attend the childbirth, do you provide him with information about labour support? |  |  |
| Q1.1.13. Is the male partner asked to leave the room for routine procedures during labour such as examination of the abdomen and vaginal examination? |  |  |
| Q1.1.14. Does your health facility inform the male partner on what to expect during his stay in the delivery room? |  |  |
| Q.1.1.15. Does your health facility organise specific antenatal classes to prepare male partners to provide support during childbirth? |  |  |
| Q.1.1.16. Does your health facility have any form of recording how many male partners attend childbirth? |  |  |

| Q1.1.17. Does your health facility provide maternity staff with educational resources related to male partners' attendance at childbirth? |  |  |
| --- | --- | --- |
| Q1.1.18. Does your health facility collaborate with any external organisations to train maternity staff about male partners' attendance at childbirth? |  |  |

# Page 3: Section 3 -Factors determining whether or not health facilities encourage male partners' attendance at labour and/or birth


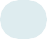


*6.*

|  | 1.Strongly disagree | 2.Disagree | 3.Neither agree nor disagree | 4.Agree | 5.Strongly agree |
| --- | --- | --- | --- | --- | --- |
| Q1.2.1. Maternity staff at this facility are not aware of the woman’s right to have a birth companion of her choice. |  |  |  |  |  |
| Q1.2.2. Maternity staff at this facility may worry that male partners would criticize the care provided to the woman during childbirth. |  |  |  |  |  |
| Q1.2.3. Our labour rooms do not offer sufficient privacy to enable male partners to attend labour. |  |  |  |  |  |
| Q1.2.4. Our delivery rooms do not offer sufficient privacy to enable male partners to attend the delivery of the baby. |  |  |  |  |  |
| Q1.2.5. The high workload of our maternity staff does not enable them to facilitate male partners to attend childbirth. |  |  |  |  |  |
| Q1.2.6. Maternity staff at this facility fear that allowing male partners to attend childbirth may spread infection to the woman and the baby. |  |  |  |  |  |

| Q1.2.7. Maternity staff in this facility have limited training on facilitating male partners' attendance at childbirth. |  |  |  |  |  |
| --- | --- | --- | --- | --- | --- |
| Q1.2.8. Our facility lacks amenities such as toilets and beds for male partners who may wish to stay overnight with their wives/partners. |  |  |  |  |  |
| Q1.2.9. Our facility does not encourage male partners’ presence at childbirth because they may become aggressive. |  |  |  |  |  |
| Q1.2.10. Staff at our facility are worried about male partners’ attendance because they fear being reported for mistreatment to an authority such the Ministry of Health. |  |  |  |  |  |
| Q1.2.11. Our health facility does not encourage male partners’ attendance at childbirth because our focus of care is the woman and the baby. |  |  |  |  |  |
| Q1.2.12. Male partners’ presence at childbirth distracts maternity staff from attending to the mother during childbirth. |  |  |  |  |  |
| Q1.2.13. Our health facility does not encourage male partners' attendance at childbirth because we consider birth companionship to be a female role. |  |  |  |  |  |

| Q1.2.14. Our health facility does not encourage male partners to attend childbirth because men are not culturally expected to be present at childbirth. |  |  |  |  |  |
| --- | --- | --- | --- | --- | --- |

Page 4: Section 4 -Health facilities' readiness to facilitate male partners' attendance at childbirth

This section asks you to report on your health facility’s readiness to facilitate male partners' attendance at childbirth*.*


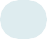


*7.*

|  | Yes | No | Do not know |
| --- | --- | --- | --- |
| Q1.3.1. Our health facility has started to encourage male partners' attendance at childbirth if it is the woman’s choice. |  |  |  |

If you answered yes to **Q1.3.1**, only answer **Q1.3.2, Q1.3.3, Q1.3.4** and **Q1.3.5.**

If the answer **to Q1.3.1** is **no** or **does not know,** answer all questions from **Q1.3.6** to **Q1.3.11.**

*7.a.*

|  | Yes | No | Do not know |
| --- | --- | --- | --- |
| Q1.3.2. Allowing male partners’ attendance at childbirth is one of our facility’s core values. |  |  |  |
| Q1.3.3. There is a high demand for male involvement in childbirth from couples attending this facility. |  |  |  |
| Q1.3.4. We face challenges in encouraging male partners to attend childbirth in our facility. |  |  |  |
| Q1.3.5. Managers of our health facility are committed to sustaining male partners' attendance at childbirth. |  |  |  |
| Q1.3.6. Our health facility is considering encouraging male partners to attend childbirth. |  |  |  |
| Q1.3.7. We believe that allowing male partners to attend childbirth will change men’s perceptions of childbirth. |  |  |  |
| Q1.3.8. We believe that implementing male partners' attendance at childbirth will enable our health facility to involve men in other safe motherhood programmes. |  |  |  |

| Q1.3.9. Introducing male partners’ attendance at childbirth will require our facility to organise specific antenatal classes for them. |  |  |  |
| --- | --- | --- | --- |
| Q1.3.10. Our health facility is ready to put in place guidelines to facilitate male partners' attendance at childbirth. |  |  |  |
| Q1.3.11. There is a plan for our health facility to make more staff available to enable our maternity unit to facilitate male partners' attendance at childbirth if it is the woman’s choice. |  |  |  |

# Page 5: Page 1

Thank for the time you provided to us in completing this questionnaire.
