# Supplementary material for: Development and early validation of questionnaires to assess system level factors affecting male partners’ attendance at childbirth in LMICs
Source: BMC Pregnancy Childbirth. 2023 Apr 17;23:258. doi: 10.1186/s12884-023-05580-y (PMC10108494; doi:10.1186/s12884-023-05580-y)
Supplement: Supplementary file 4 — Additional file 4. QUESTIONNAIRE FOR MATERNITY STAFF. [file 12884_2023_5580_MOESM4_ESM.pdf]

QUESTIONNAIRE FOR MATERNITY STAFF

Page 1: ABOUT THIS STUDY

1. Participant Number (Leave the space blank, to be completed by the data collection team)

1.a. ....../....../2019

Dates need to be in the format 'DD/MM/YYYY', for example 27/03/1980.

(dd/mm/yyyy)

Male partners' attendance at childbirth in Rwandan health facilities: current practice, service level factors, and health providers' views.

Dear Participant,

You are invited to participate in our study titled 'Male partners' attendance at childbirth in Rwandan health facilities: current practice, service level factors, and health providers' views'.

As you will have read in the Participant Information Sheet provided to you, your participation in this study will contribute to obtaining preliminary data on perceptions and attitudes regarding the acceptability and feasibility of male partners' attendance at

childbirth in Rwandan health facilities. Your participation will provide data about the extent to which maternity staff in Rwandan health facilities facilitate male partners' attendance at labour and/or birth. It is expected that information from this study may inform policy on male partners' attendance at childbirth if it is the woman's choice. It may also inform interventions targeting barriers to women's choice of birth companion.

Once again, we thank you for taking time to complete this questionnaire.

Consent to participate in the study

You are not required to sign a separate consent form. You will confirm your voluntary participation in the study by ticking **Yes** or **No** below. Once you agree to participate in the study, the return of completed questionnaires will be taken as consent.

2. I am interested to participate in this study.

- ○ Yes
- ○ No

2.a. If you opted to participate in the study, do you feel comfortable to read and answer all questions in this questionnaire in English language?

- ○ Yes
- ○ No

If the answer is no, do not continue to the next stage of this survey. Thank you for taking your time to participate in our study.

The questionnaire comprises five sections:

In section 1, you are asked to provide your demographic background information.

Section 2 asks you to report about your practice regarding the facilitation of male partners' attendance

at childbirth.

Section 3 asks you to to rate the extent to which you agree or disagree with statements about perceptions of male partners' attendance at childbirth based on your experience in caring for expectant women and their families.

Section 4 asks you to rate the extent to which you agree or disagree with statements about the acceptability of male partners' attendance at childbirth based on your interaction with the expectant women and their families.

Section 5 asks you to rate the extent to which you agree or disagree with statements about feasibility of encouraging male partners' to attend childbirth based on your experience in providing care to the mothers during labour and childbirth.

Page 2: Section 1: Demographic information

This section asks you some demographic information about you.

3.

	1. Public Health Centre	2. Public District Hospital	3. Public Referral Hospital	4. Private clinic or polyclinic	5. Private Hospital
1. Please tick one name used to describe the facility you work for.	☐	☐	☐	☐	☐

3.a.

	18-24 years old	25-34 years old	35-44 years old	45-54 years old	55 years or older
2. What is your age?	☐	☐	☐	☐	☐

3.a.i.

	Male	Female	Prefer not to say
3. To which gender do you most identify with?	☐	☐	☐

3.a.i.a.

	Midwife	General Nurse	Obstetrician	Medical Doctor	Other (Please specify):..

4. Which category best describes your role in maternity care or maternity ward?	☐	☐	☐	☐	☐

3.a.i.a.i.

	Secondary school	Diploma	Bachelor's degree	Master's degree	Doctorate degree	PhD
5. What is the highest level of education you have attained?	☐	☐	☐	☐	☐	☐

3.a.i.a.i.a.

	6-11 months	1-2 years	3-5 years	6-10 years	10+years
6. How long have you been working worked in the maternity ward?	☐	☐	☐	☐	☐

Page 3: Section 2 -Practices towards encouraging male partners' attendance at childbirth

4. This section consists of items related to what maternity staff may do to encourage and support male partners when they attend labour and/or birth. Please read each item carefully and indicate how often you practise that.

	1.Never	2.Sometimes	3.Frequently	4.Always
Q2.1.1. I develop a birth plan with the couple indicating the level of the male partner's participation during childbirth.	☐	☐	☐	☐
Q2.1.2. I ask the woman if she wants to have her male partner present at labour.	☐	☐	☐	☐
Q2.1.3. I ask the woman if she wants to have her male partner present at birth.	☐	☐	☐	☐
Q2.1.4. I respect the woman’s choices regarding the presence of her male partner during childbirth.	☐	☐	☐	☐
Q2.1.5. I insist that any male partner who accompanies the woman to give birth should stay with her throughout childbirth period regardless of the preference of the woman.	☐	☐	☐	☐
Q2.1.6. I ask the woman's male partner if he wishes to attend childbirth.	☐	☐	☐	☐
Q2.1.7. I permit the woman's male partner to stay with the woman during childbirth.	☐	☐	☐	☐

Q2.1.8. I teach the woman's male partner how to support her to adopt comfortable positions during labour.	☐	☐	☐	☐
Q2.1.9. I teach the woman's male partner how to provide her with emotional support during labour.	☐	☐	☐	☐
Q2.1.10. I teach the woman's male partner how to massage her during labour.	☐	☐	☐	☐
Q2.1.11. I seek the woman’s consent before informing her male partner about the care provided to her during childbirth.	☐	☐	☐	☐
Q2.1.12. I communicate to the woman's male partner about her childbirth progress.	☐	☐	☐	☐
Q2.1.13. I involve both parents in any decisions regarding the care provided to the woman throughout childbirth.	☐	☐	☐	☐
Q2.1.14. I listen to the male partner's concerns about the woman’s condition during childbirth.	☐	☐	☐	☐
Q2.1.15. If a woman's male partner becomes distressed, I ask him to leave the room.	☐	☐	☐	☐
Q2.1.16. I ask the woman's male partner if he wants to watch the baby being born.	☐	☐	☐	☐

Page 4: Section 3 -Perceptions about male partners' attendance at labour and/or birth

5. **The following section comprises of items relating to the perceptions about male partners’ attendance at childbirth. Please read each statement carefully and indicate the extent to which you agree with the statement.**

	1.Strongly disagree	2.Disagree	3.Neither agree nor disagree	4.Agree	5.Strongly agree
Q2.2.1. Male partners' presence at childbirth is one of the ways to make childbirth care more family centred.	☐	☐	☐	☐	☐
Q2.2.2. A woman's male partner must not be allowed inside the maternity ward if the woman has a female birth companion.	☐	☐	☐	☐	☐
Q2.2.3. Male partners should attend childbirth to witness what happens during childbirth.	☐	☐	☐	☐	☐
Q2.2.4. Allowing male partners to attend childbirth protects healthcare providers against any allegations of negligence.	☐	☐	☐	☐	☐
Q2.2.5. A woman's male partner's presence makes her feel less stressed during childbirth.	☐	☐	☐	☐	☐

Q2.2.6. A woman's male partner provides verbal reassurance to her during labour.	☐	☐	☐	☐	☐
Q2.2.7. A woman's male partner's attendance at birth fosters his bonding with the baby.	☐	☐	☐	☐	☐
Q2.2.8. Male partners need to attend childbirth to develop a better appreciation of the woman.	☐	☐	☐	☐	☐
Q2.2.9. A woman's male partner may assist her to do breathing and relaxation exercises.	☐	☐	☐	☐	☐
Q2.2.10. Male partners’ presence may impact on couples’ subsequent pregnancy planning.	☐	☐	☐	☐	☐
Q2.2.11. A woman's male partner's presence can make her feel uncomfortable during labour.	☐	☐	☐	☐	☐
Q2.2.12. Male partners' presence during delivery can negatively impact on couples' sexual relationships.	☐	☐	☐	☐	☐
Q2.2.13. The male partner's presence can be detrimental to the wellbeing of the labouring woman.	☐	☐	☐	☐	☐

Q2.2.14. A woman's male partners' presence may provoke undesirable emotional reactions from her during childbirth.	☐	☐	☐	☐	☐
Q2.2.15. The woman's male partner may faint during the delivery of the baby.	☐	☐	☐	☐	☐
Q2.2.16. Permitting male partners' presence at childbirth can lead to congestion in the maternity ward.	☐	☐	☐	☐	☐
Q2.2.17. Male partners' presence at childbirth will increase stress on healthcare providers.	☐	☐	☐	☐	☐
Q2.2.18. Maternity staff may feel uncomfortable when they are watched by male partners while offering care to the women during childbirth.	☐	☐	☐	☐	☐
Q2.2.19. A woman's male partner's presence does not help at all in the care of the woman during childbirth.	☐	☐	☐	☐	☐

Page 5: Section 4 -Attitudes regarding the acceptability of male partners' attendance at labour and/or birth

6. This section consists of items regarding the acceptability of male partners' attendance at childbirth. Please read each statement carefully and indicate the extent to which you agree or disagree with the statement.

	1.Strongly disagree	2.Disagree	3.Neither agree nor disagree	4.Agree	5.Strongly agree
Q2.3.1. It is acceptable to allow a male partner to be present during labour if it is the woman's choice.	☐	☐	☐	☐	☐
Q2.3.2. It is acceptable to allow a male partner to be present at birth if it is the woman's choice.	☐	☐	☐	☐	☐
Q2.3.3. It is acceptable to oblige male partners to stay with the woman throughout childbirth once they arrive at the place of delivery.	☐	☐	☐	☐	☐
Q2.3.4. It is acceptable to only allow the woman's male partner to stay with the woman during delivery.	☐	☐	☐	☐	☐
Q2.3.5. It is acceptable to only allow the woman's male partner to stay with the woman if she is expected to deliver normally.	☐	☐	☐	☐	☐

Q2.3.6. It is acceptable to allow all male partners to attend childbirth without imposing any limitation on them.	☐	☐	☐	☐	☐
Q2.3.7. It is acceptable for the maternity staff to facilitate a woman's male partner to attend childbirth to share the birth experience with the woman.	☐	☐	☐	☐	☐
Q2.3.8. It is acceptable to allow the woman's male partner to stay with the woman to offer practical support to her such as massaging her back during labour, holding her hand during labour, offering her food and drink, and encouraging her to cope with labour.	☐	☐	☐	☐	☐
Q2.3.9. It is acceptable for healthcare providers to allow the woman's male partner to take pictures and videos throughout childbirth.	☐	☐	☐	☐	☐
Q2.3.10. It is acceptable for healthcare providers to assist the woman's male partner to support her during childbirth.	☐	☐	☐	☐	☐

Q2.3.11. It is acceptable to allow the woman's male partner to attend Caesarean section.	☐	☐	☐	☐	☐
Q2.3.12. It is acceptable for health providers to establish a good relationship with the woman's male partner to enable him to cope with her labour.	☐	☐	☐	☐	☐
Q2.3.13. It is acceptable to not allow male partners who look anxious to stay with the woman during childbirth.	☐	☐	☐	☐	☐

Page 6: Section 5-Attitudes regarding the feasibility of male partners' attendance at labour and/or birth

7. **This section consists of items regarding the feasibility of male partners’ attendance at childbirth. Please read each statement carefully and indicate the extent to which you agree or disagree with the statement.**

	1.Strongly disagree	2. Disagree	3.Neither disagree nor agree	4. Agree	5. Strongly agree
Q2.4.1. It is feasible for me to advise women's male partners about coping mechanisms to adopt during childbirth.	☐	☐	☐	☐	☐
Q2.4.2. It is feasible for me to inform the woman of her right to choose her male partner as her birth companion.	☐	☐	☐	☐	☐
Q2.4.3. It is feasible for me to ask the woman's male partner if he wishes to attend childbirth.	☐	☐	☐	☐	☐
Q2.4.4. It is feasible for me to inform the woman's male partner what happens during labour and birth when he attends antenatal appointments.	☐	☐	☐	☐	☐
Q2.4.5. It is feasible for me to permit the woman's male partner to be with her regardless of the type of birth.	☐	☐	☐	☐	☐
Q2.4.6. It is feasible for me to teach the woman's male partner how to support her to adopt comfortable positions during labour.	☐	☐	☐	☐	☐

Q2.4.7. It is feasible for me to show the woman's male partner how to massage her during labour.	☐	☐	☐	☐	☐
Q2.4.8. It is feasible for me to help male partners articulate their needs during their stay in the maternity ward.	☐	☐	☐	☐	☐
Q2.4.9. It is feasible for me to ask the woman's male partner to leave the labour room if he becomes distressed.	☐	☐	☐	☐	☐
Q2.4.10. It is feasible for me to listen to the woman's male partner's concerns about the woman’s condition during childbirth.	☐	☐	☐	☐	☐
Q2.4.11. It is feasible for me to show the woman's male partner where to sit while staying in the labour ward.	☐	☐	☐	☐	☐
Q2.4.12. It is feasible for me to welcome the woman's male partner in the delivery room to welcome the baby.	☐	☐	☐	☐	☐
Q2.4.13. It is feasible for me to thank the woman's male partner for his efforts to be with the woman during childbirth.	☐	☐	☐	☐	☐

Page 7: Final page

Thank for the time you provided to us in completing this questionnaire.
